# Domestic Intelligence

**Published:** 1800-01

**Authors:** 


### *Domestic Intelligence.*

THE Vaccine Pock Inoculation has been found by many practitioners to produce, in some instances, eruptions even resembling those of the Small Pock; and the matter of those cows has been also found to produce similar eruptive cases. Hence, in practice, the rule has been, since these observations were made, to avoid inoculating with the matter of patients who had such eruptions. Different opinions have been entertained as to the causes of these eruptions, and the occurrence of them has occasioned the credit of the Cow-pock Inoculation to suffer considerably in the opinion of many persons. Dr. PEARSON has favoured us with a paper on this part of the History of the Cow-pock, in which he estimates the value of the new practice, on the supposition that such eruptive cases are unavoidable in a certain proportion. The paper came too late for insertion in this number, but will be given in our next. In the mean time, we have the satisfaction to announce, that these cases do not reduce the value of the Cow-pock Inoculation in a great degree, and that advantages unexpected are now manifest, which more than counter balance the disadvantage of eruptions.

Dr. BRADLEY will commence his Spring Course of Lectures on the Theory and Practice of Medicine, on Monday, January 20th, at the Lecture-Room, No. 102, Leadenhall-street, at Six o'Clock in the Evening. Terms, Three Guineas.

Mr. PEARSON will commence his Spring Course of Lectures on Surgery, at his house in Golden-Square, on Monday, January 20th, at Seven o'Clock in the Evening.

Dr. MOSSMAN, of Bradford, Yorkshire, has, for several years, been employed in writing an "Essay to elucidate the Nature, Origin, and Connection subsisting between *Scrophula* and *Glandular Consumption*; including, a Brief History of the Effects of *Illnley Spa*; with Observations on the Medicinal Powers of *Digitalis*; and Strictures on some Opinions relative to that Plant."—We are, however, authorised to observe, that this Essay will contain no cases of the effects of Digitalis in Pulmonary Consumption; as Dr. Mossman will obligingly continue to communicate them through the medium of our Journal, and has promised to transmit to us several very remarkable cases, for our next Number.

We understand that the Eighth Volume of "Medical Facts and Observations, by Dr. SIMMONS," is now in the Press, and will be published toward the end of January, or early in February next.

CRITICAL

